# Human Temperaments. VI.—The Man of Business


**Published:** 1916-07-08

**Authors:** 


### HUMAN TEMPERAMENTS.

### VI.—The Man of Business.*

The business temperament is the contradictory opposite of the artistic temperament. There are plenty of men of artistic temperament in business, and a sad hash they make of it if the temperament is much developed, and there are artists who have the business temperament in addition to that of the artist. The business temperament is quite compatible with the temperament of the artist, but quite incompatible with the artistic temperament.

The man of business temperament is he who extracts the maximum of benefit from his circumstances. He need not be clever, though to attain great success some cleverness is, of course, necessary, but he is eminently capable. Nothing is more characteristic of the business man than his power of discerning the main point and of sticking to it. Out of a tangled maze of facts, factors, and considerations he seizes unerringly upon that which is of most importance to the purpose in view, and never loses sight of it, never allows himself to be diverted from it. Subordinate tasks have to be undertaken, subsidiary problems must be solved, preparatory arrangements must be made, but the man of business temperament never allows himself, as the merely clever person so often does, to allow the contemplation of the means to obscure his vision of the end, or to allow that which was undertaken as a means to some ulterior end to become an end in itself and for itself. The clever surgeon, in devising the complicated details of an operation, and thinking out in every aspect the best way to perform it, is apt to forget to ask himself the question, Is this operation necessary in this case or not? Is it better to treat this patient by operation or by some other means? The operation is not an end in itself. Even to perform the operation successfully is not the main end to be pursued. The main end is to cure the patient; and this is the end that the surgeon of business temperament will never lose sight of, but that the merely clever surgeon is very apt to lose sight of.

Promptitude is another prominent characteristic of the business temperament, and one which contrasts sharply with the dilatory and procrastinating habit of the artistic temperament. The man of business temperament is prompt in all his doings. His letters are answered by return of post. His accounts go out and his bills are settled to the day. Whatever task presents itself is taken in hand at once. When Napoleon, who was the supreme example of the business temperament, heard of Sir John Moore's dash upon his communications, he started from Madrid within the hour. But it is not only in action that the man of business temperament is prompt. He is prompt in decision also. While the artistic temperament is wondering what had better be done, hesitating and vacillating, making up and unmaking its mind, the business temperament has decided what to do and done it. This facility of prompt decision rests upon the fundamental business quality of discerning and selecting without fail the main end to be striven for. When this is held in view, a multitude of alternative courses are swept aside as irrelevant. They may be important, but their importance is subsidiary. The business temperament looks to what is vital, and from the constant habit of selecting the thing that is vital acquires a readiness to recognise it and disentangle it from things that are important only, and not vital. Hence its choice becomes cleared. Instead of being among a dozen things, it is limited to two or three alternatives; and between these the choice of that which most contributes to the main and vital end is more easily, and therefore more promptly, made. With the business temperament promptness of decision no less than promptitude of action becomes a habit.

From the same clear view of the main purpose to be pursued, it follows that the man of business temperament is persevering. The fixation of his attention and interest on the main purpose is a safeguard against wandering efforts. He gets through what he is doing, and sticks to it until it is done, for the same reason that the traveller keeps to the road, and does not stray into the adjoining fields and woods; that is to say, because he looks to the end in view. He never forgets that the subsidiary task on which he is engaged is subsidiary. He does not stray aimlessly from the road because the adjoining country is more inviting, but if he sees a path that will take him more directly to his destination, he quits the road instantly and follows the new path. He is shy of novelties only when he does not see clearly that they serve his main purpose, that they lead to his main end; but he is quick to see and adopt them when they do so. The business man is not often clever at devising new expedients. That is not his business. His business is to examine the expedients proposed by clever men, and to decide whether they will or will not serve his purpose better than those he is using. If they will not, it matters not how clever they are, he will have nothing to do with them. If they will, he sacrifices his existing arrangements without hesitation and without scruple.

The man of business temperament is a good organiser; that is to say, he is orderly not only in his acts, but in his thought. He does not deal with things in the gross, but arranges and classifies them; and again his power of organisation depends on his power of seeing clearly and adhering closely to the main purpose. For the same things can be classified in as many ways as there are purposes to serve by the classifications, and the mode of classification should be governed entirely by the purpose it has to serve. The gardener classifies plants

* The previous articles appeared on May 6, 13, 27, and June 10 and 24, 1916; and a letter on the subject on May 20.

primarily according as they are ornamental or edible, for his purposes are to make one part of the garden ornamental, and to grow edible plants in another. The botanist classifies plants according to their mode of reproduction, for his purpose is to discover their natural affinities. The pharmacist classifies them according to their effects on those who consume them, for his purpose is to produce these effects. The economist classifies them according to their utility and merchantableness, in accordance with his purpose of growing them to a profit. When we say that a man is a good organiser, we mean that he is a good classifier for the purpose he has in view. If he organises a business, he classifies his goods or his operations of manufacture into departments, and he is a good or a bad organiser according as his classification serves well or ill the purpose he has in view. In a factory he must so classify his departments that his raw material pursues the easiest and most uninterrupted course from one department to another until it ends as a finished product at the place from which it is easiest to deliver it. He must organise his staff so that he can lay his finger on the particular man who is responsible for any particular operation, and so that every man looks to but one superior for orders, and has full control subject to those orders. He classifies each kind of work into a separate department with a separate staff, and each staff into its own hierarchy of officials. From beginning to end, organisation is classification, and as classification always has reference to purpose and is good or bad according as the purpose is kept in view, and as it serves the purpose, he is the best organiser who keeps the purpose of the organisation most steadily in view.

In organising or classifying men for any purpose it is very necessary not only that the classification should suit the purpose in view, but that the men also should be fit for the places they take in the organisation. To put round men into square holes, and *vice versâ*, is bad organisation. The man of business temperament, who is a good organiser, never does this. In order that he should avoid this mistake he must estimate with accuracy not only the qualities that are required for the place, but also the qualitics possessed by the man he proposes to put into the place. In other words, he must have an insight into character. This is the inborn gift of the business temperament. Steady fixation of the attention on purpose may be cultivated; promptness of action and of decision may develop by exercise; classification may be learnt; but insight into character is inborn, and he who has it not as a birthright can never acquire it in any considerable degree. All of us possess some rudiment of it, no doubt, and some qualities show themselves so conspicuously that there is little difficulty in gauging them; but to tell by a single interview whether or not a man is trustworthy, as Charles Darwin's father could, or whether he has the moral courage to bear great responsibilities, or whether he is of prompt decision and clear judgment in important matters, this is a gift which few possess, and which perhaps none can acquire. Anyone can tell on a very short acquaintance whether a man is clever or stupid, conceited or modest, reticent or leaky, strong or weak in character, for these are qualities that blaze out conspicuously, that shout aloud, and cannot be overlooked, but by what fine and subtle indications, elusive to the eye and ear, and discernible only by some sixth sense, it can be discovered whether a man is trustworthy, loyal, courageous, ready in emergencies, capable of inspiring confidence, of leading and managing men, of devotion to a cause and of steadiness in adversity—these are to most people unknown, but they are in some mysterious way perceived by the man of business temperament, whose most valuable faculty is his ability to read character aright, and to place round men in round holes and square men in square holes.

